# Reducing Low Birth Weight among African Americans in the Midwest: A Look at How Faith-Based Organizations Are Poised to Inform and Influence Health Communication on the Developmental Origins of Health and Disease (DOHaD)

**DOI:** 10.3390/healthcare5010006

**Published:** 2017-02-04

**Authors:** Crystal Y. Lumpkins, Jarron M. Saint Onge

**Affiliations:** 1 Department of Family Medicine, University of Kansas Medical Center, Kansas City, KS 66106, USA; 2 Department of Sociology, Health Policy and Management, University of Kansas-Lawrence, Lawrence, KS 66045, USA; jsaintonge@ku.edu

**Keywords:** low birth weight, African Americans, faith-based organizations, developmental origins of health and disease/DOHaD, health promotion, health communication, media advocacy, social marketing

## Abstract

Low birth weight (LBW) rates remain the highest among African Americans despite public health efforts to address these disparities; with some of the highest racial disparities in the Midwest (Kansas). The Developmental Origins of Health and Disease (DOHaD) perspective offers an explanation for how LBW contributes to racial health disparities among African Americans and informs a community directed health communication framework for creating sustainable programs to address these disparities. Trusted community organizations such as faith-based organizations are well situated to explain health communication gaps that may occur over the life course. These entities are underutilized in core health promotion programming targeting underserved populations and can prove essential for addressing developmental origins of LBW among African Americans. Extrapolating from focus group data collected from African American church populations as part of a social marketing health promotion project on cancer prevention, we theoretically consider how a similar communication framework and approach may apply to address LBW disparities. Stratified focus groups (*n* = 9) were used to discover emergent themes about disease prevention, and subsequently applied to explore how faith-based organizations (FBOs) inform strategic health care (media) advocacy and health promotion that potentially apply to address LBW among African Americans. We argue that FBOs are poised to meet health promotion and health communication needs among African American women who face social barriers in health.

## 1. Introduction 

### 1.1. Low Birth Weight and DOHaD among African Americans

Racial disparities in low birth weight (LBW) are pervasive among minority populations, especially among African Americans (AA) [1]. For example, the percentage of low birthweights of all births for AA is nearly double that of non-Hispanic whites [2]. Currently, Kansas has the highest infant mortality rates for non-Hispanic blacks in the U.S. (14.8), with Missouri also rating poorly (12.4) [3]. Additionally, AA have nearly double the low birth weight rates of non-Hispanic whites in both Kansas and Missouri [2]. While national and local public health promotion efforts to address these problems have made contributions to improve maternal and infant health among AAs, LBW disparities remain a public concern. The long lasting implications of LBW coupled with the persistent challenges of inequality and cultural barriers highlights the potential role of a community directed health communication approach that incorporates the developmental origins of health and disease (DOHaD) perspective [4]. Specifically, we draw on data from a sample of focus groups with AA women from the bi-state Kansas City metropolitan area to argue that a strong community-focused social marketing approach aimed at fostering trust and long-term health integration through established faith-based organizations (FBOs) may be a fruitful approach to addressing LBW among AA women.

FBOs are trusted, affinity organizations that serve as health promotors and health communicators within a community. Inequities in health outcomes such as LBW are exacerbated by inequitable health information among minority populations [5]. Through a social marketing approach, FBOs can help to initiate and maintain health communication strategies that are dynamic, multi-faceted and relatable to communication disadvantaged (e.g., AA female) populations. Dynamic health communication programs can offset social determinants of health (SDoH) (e.g., poverty, discrimination, barriers to care, etc.) through culturally relevant and digestible messages that are targeted to specific segments of the population. These programs may be designed to identify not only attitudes, perceived norms and self-efficacy but also behavioral beliefs to also facilitate positive health behaviors and coping strategies among AA. Messages that offset the SDoH are relatable, understandable (relatively easy to process) and align with existing cultural beliefs and ideology. Capturing and incorporating these determinants (attitudes, perceived norms, and self-efficacy) [6] that AA have toward FBOs into LBW messaging may prime or persuade individuals to perform a positive behavior. 

Early childhood LBW may have long lasting health implications and lead to adult racial health disparities [7]. Risk factors for LBW include both genetic [8] and social explanations [9,10], with a general understanding that social inequalities shape life experiences and environmental exposures to risk. For example, research has shown that native-born AA have a higher chance of LBW compared to foreign-born AA [11] highlighting the role of social and environmental exposures on maternal and fetal health. Indeed, LBW is concentrated in more disadvantaged groups, with unmarried, low educated, AA mothers as one of the most marginalized groups in the U.S with the highest rates of LBW [12]. 

Although the behavioral factors that increase LBW have become more well-known (e.g., prenatal care, maternal smoking, nutrition), the challenges to limiting the impact of social determinants endure. Social disadvantage has cumulative impacts throughout the life course with the long-term effects of LBW potentially becoming worse over time, resulting in increased health disparities. For example, while medical advances continue to increase survival rates from LBW, the same social factors that increased risk to LBW remain (i.e., poverty), which may increase and compound risk whereby children born LBW may receive fewer and less effective investments in post-natal care and remain exposed to health-compromising, disadvantaged environments [12,13]. Exposures to disadvantage, such as poverty, barriers to care (e.g., lack of insurance, perceived discrimination, distrust in medical interventions) and stress remain concentrated among disadvantaged minority populations, requiring an emphasis on dynamic health communication programs with the potential to offset social determinants of health. 

### 1.2. DOHaD Communication within a Social Marketing Framework 

Traditional public health promotion has helped to mitigate barriers among AA populations through disease prevention promotion and health communication programs but gaps remain because messages and delivery of current public health efforts are often not transferrable to the target culture, are devoid of existing policies designed to protect and or promote health equity and are focused on downstream factors [14]. While traditional efforts have largely taken an epidemiologic approach, reflecting dominant societal views, values and culture, a social marketing (SM) framework incorporates a more adaptive and dynamic approach. Social marketing, a concept developed in the early 1970s [15] is an approach that many health communication researchers and practitioners employ to impact behavior change. Social marketing principles and strategies are grounded in the marketing mix (Product, Price, Promotion and Placement) whereby these factors are used to guide strategies to persuade behavior change among the target audience about a given pro-social topic [16]. Embedded in the principles of SM however, is the capability of segmentation where specific pro-social behaviors (i.e., intangible products of health such as screening for certain diseases) are promoted to segments of the population; and also thus presented as opportunities for social change within a public health context [17]. Segmentation allows the marketer to identify and utilize shared values, beliefs and lifestyle factors and subsequently use these elements to impact behavior. Here, segmented health information about LBW is transferred, disseminated and socially marketed through a relatable, authentic and appealing channel to AA women within FBO populations. 

New and emerging research shows the promise of employing dynamic communication similar to SM that informs and translates DOHaD “findings from the bench to population-level health promotion and disease prevention [14] (p. 170)”, with the potential to intervene at multiple levels to address broad health inequities such as LBW. 

DOHaD communication combined with SM principles that integrate community and societal partners move beyond single-level communication. Impacting public health efforts through a DOHaD communication and SM framework addresses the public health issue of LBW on multiple levels and has the potential to incorporate multifaceted views of not only the traditional communicators (e.g., the medical establishment) involved in the communication process but less-dominant individuals who are from within segments of the population. Community organizations, such as FBOs, that are trusted and influential among AA have the capacity to impact public health outcomes through organizationally provided social support. These organizations have traditionally been champions for vulnerable populations; they are also demonstrated health promotors and health communicators for numerous health issues and have been instrumental in helping to impact health behavior and improve public health [18,19]. Extrapolating from focus group data collected from African American church populations as part of a social marketing health promotion project on cancer prevention, we theoretically consider how a similar communication framework and approach may apply to address LBW disparities. 

### 1.3. Aims

We aim to determine how FBOs are poised to meet public health communication needs among AA women. Building on previous results from a social marketing health promotion project involving AA church populations in the Kansas and Missouri bi-state area [20], we draw parallels to a cancer initiative to theoretically consider how a similar communication framework and approach may apply to LBW disparities. Specifically, we draw from a sample of AA women to consider two primary ways that FBOs can serve to address risk factors for LBW. First, we consider the role of SM principles within a DOHaD communication framework. Second, we identify key conceptual components of strategic media advocacy through SM segmentation among AA women and consider the integration of FBOs during communication about LBW risk factors to combat developmental health disparities among an underserved population.

## 2. Method

### 2.1. Research Setting and Participants

Data collection took place at churches in Kansas and Missouri, over an 8-month period in 2012 and was approved by the university’s human subjects committee (project identification code number 00000316; approval #CR00002398). Women who participated were part of a larger Community-Based Participatory Research (CBPR) study that explored attitudes, beliefs and behavior intention toward colon cancer prevention and perceptions of FBOs as marketers of cancer prevention health promotion among AA church populations. The present study sample included 9 focus group interviews (*n* = 78) that were stratified by state (Kansas or Missouri); area of the city (urban or suburban) and by gender (male and female). The sample was purposive and drawn from a group of 124 AA women from 14 churches who participated in the parent study. Women who were included in the sample were AA, between the ages of 35 and 70 who either attended church or lived in the bi-state areas of Kansas and Missouri (see Table 1). Participants were recruited by FBO community members and university staff over a 12-month period during church services and community events. After recruitment, women were invited to a scheduled discussion and asked to provide consent and also fill out a demographic survey prior to the focus group discussion. They were given both a meal and instructions about the focus group protocols prior to participating in a 90-minute focus group led by university staff. All participants were compensated $20 for their time.

Focus groups were guided by pre-determined topical sections yielded through previously conducted community-based participatory meetings with FBO laypersons, researchers and members from the AA community. Focus group questions pertaining to respondent views about barriers and facilitators to health care and health care access; cancer risk and prevention; and the role of an FBO (i.e., church) as a social marketer of health promotion of disease prevention were included. 

Authors applied emergent themes from the focus groups to explore how DOHaD communication within a social marketing framework has potential applications to issues of LBW. 

### 2.2. Analysis 

The authors took a multi-method approach to interpreting, analyzing and coding data collected through focus group discussions. Three individuals followed an open coding process where each coder read transcripts multiple times to look for emerging themes among five topic areas; these were pre-determined through work with a community advisory board and were slightly modified based on feedback after the first set of focus groups. The topic areas stayed the same throughout data collection and included: Experiences with Cancer; Experiences with Colon Cancer; Colon Cancer Prevention, Diagnosis and Treatment; as well as the two primary areas of focus for this study: Barriers to Screening; and Faith-Based Prevention Messages. Some questions included: How does cost figure into whether or not to get screened? How comfortable would most people be with bringing these things (screening) up with their doctor? What do you think is most effective in getting cancer prevention messages across to church members? How can the church be used to transmit health prevention messages? What is your opinion of the church serving as a sponsor of cancer prevention promotion materials?

Coders were from diverse backgrounds and included two AA females (one faculty member and one medical student) and an Indian female (post-baccalaureate student). The diversity of views and perspectives addressed validity and reliability of the coding and the coding process. Coders met two times over the course of four months to compare and contrast categories discovered during their individual open coding process. The open coding process and subsequent contrast and comparison enabled contextualization and categorization of data. The primary and secondary author subsequently took these results to apply a grounded theory approach to explore how women interpreted FBOs as social marketers of disease and prevention among church populations. While grounded theory traditionally occurs when the researcher collects data and analyzes the data [21], here it was applied as an analytical lens to explore how AA women constructed and symbolically interpreted [22] FBOs as SM of disease prevention following initial coding of data. 

The authors drew parallels between cancer health disparities and LBW and also well-documented barriers and facilitators to proactive health behavior among AA women. 

#### Focus Group Results

The following three salient themes emerged about health care access, cost and FBOs as SM within a DOHaD context:

*FBOs Help* *Reduce Medical Cost Worry*

The first salient theme among the sample was the worry of health care costs. In some cases women indicated that they had to forgo paying for preventive care to purchase grocery and or clothing for their family to avoid the burden of medical bills. It was important that they also had utilities paid and rent before taking care of other preventive medical needs.

*(Participant from Church 7)—“I have to weigh the cost of screening with costs of providing for others, medicine,* etc. *Screening becomes low priority and takes a back seat.”*

The undue burden of cost worry among this population has been a perpetual barrier and contributes to inadequate healthcare over the life course. Among AAs, the cost of healthcare and related barriers have presented persistent challenges and increased vulnerability; the inequitable status begins in infanthood and is perpetuated throughout life. Addressing cost through FBOs within a DOHaD communication framework may mitigate some of these barriers to existing views of the “high” cost of care and living well. Moreover, rather than considering health care and general situational challenges as separate costs, clearer, directed and segmented communication could help to clarify the importance of how both current health conditions and stressors both increase risk of LBW.

*FBOs are Trusted* *Marketers of Preventive Information*

Participants were provided with a definition of social marketing and an explanation of what it means to be a sponsor. They were subsequently asked pointed questions about whether they saw FBOs as a social marketer of health and who they thought was the most trusted individual within the organization to deliver this type of health information (i.e., cancer prevention).

*(Participant at Church 4) “I think that anything* *that your church sponsors or are participating in that you would want to participate in it.”*

*(Participant at Church 1) A testimony is a greater sales pitch than* *a regular commercial.”*

FBOs Are Trusted Networks of Health Information

SM principles applied within a DOHaD perspective involves the delivery and deliberate strategic messaging by trusted organizations and individuals to enhance the promotion of DOHaD. Currently, DOHaD is visible in some health promotion/communication programs but only a few programs incorporate trusted entities into the creation *and* delivery of the health information. The opportunity to take pertinent health information and to strategically place pro-health, pro-social ideas and suggested health behavior within trusted, affinity networks has potential to bolster appeal, increase positive perceptions and reduce suspicion and non-trustworthy communication and endorsement.

(Participant from Church 2) “Our pastor’s endorsement is important because he’s our leader, the protector of our souls and we believe him.”

*(Participant at Church 5) “The more you hear health messages,* *the more you are likely to listen,”*

*(Participant from Church 7) “You know I asked (Unnamed Cancer Organization) to come over here for the health fair, okay and I asked them to come over and do screenings in a black neighborhood for a black church, black woman on the telephone asking, ok? I was turned down cold.* *Turned straight down… I was over in Mission, Kansas (relatively White, affluent area) and they were doing it over there. You see what I’m saying?”*

## 3. Discussion

### Communication Implications and Practical Considerations 

The strategic incorporation of social marketing principles into health communication research, practice and programming has far reaching implications for AA concerning LBW. Salient themes among the sample show FBOs are trusted marketers of disease prevention and are also seen as gatekeepers of pertinent public health information; FBOs were seen as trusted entities to broker positive and/or life-threatening information. The sample also overwhelmingly looked to pastors within these FBOs as credible voices for health promotion. Applied to a DOHaD perspective, FBOs are positioned for promulgating health information about LBW throughout several phases of life for the mother and infant. Secondly, key conceptual components were identified for strategic media advocacy through SM segmentation among AA women. Pinpointing control and/or behavioral beliefs about worry of cost of care, trust in the health communicator and also trust in the *type* of health information may be building blocks for integration of FBO marketed and tailored information about LBW risk factors. Incorporation of these factors into health communication programs increase the probability of combating developmental health disparities among an underserved population.

The primary limitation of this research was the sample focus. The sample ranged in ages outside of primary childbearing years (between 35 and 70 years of age) with primary queries focused on cancer prevention and faith-based messages from FBOs. Nonetheless, we found the drawing on this sample allowed us to theoretically apply findings to broader issues of cumulative disadvantage and identified similar challenges applicable to LBW. Future research would benefit from interviews specifically pertaining to LBW. Through additional analyses in the context of LBW, we identified overarching themes of fears, held beliefs and barriers. While clearly not the same, the issues of cancer and LBW disparities are both prevalent among this population and may occur and impact AA women over the lifespan. In fact, several younger women in this sample referred to reproductive screening (albeit, not a primary theme) when asked about health care access and preventive screening. Women expressed that routine screening that began as a youth, was a common and expected practice but not always welcomed. 

Public health campaigns designed to reach vulnerable populations with DOHaD elements are either scarce or are not strategically woven into messaging strategies and delivery of health information. These deficiencies often derive from poor or little planning that excludes culturally relevant factors for the target audience and multiple avenues to effectively communicate positive health behavior change. Public health campaigns that harness communication science to optimize reach and impact will be the most successful [23,24]. Using a SM framework to communicate DOHaD of LBW among AA addresses the social, environmental and policy challenges to reduce disparities. Viswanath and Bond [25] argue the importance of the broader social environment in understanding the role of communication on health behaviors. Specifically, they suggest that social determinants moderate the influence of health communications. As we found, FBOs are a means to overcome barriers such as low-SES through social integration and increasing receptiveness within a trusted organizational and cultural context. 

FBOs have historically and traditionally been shown to influence opinions and behaviors among AA. As demonstrated, SM serves as a useful tool utilized by community organizations to appeal to AA as trusted health communicators of prevention and health behavior. By addressing key components of SM (such as segmentation), FBOs have the ability to strategically communicate effective coping strategies to reduce both LBW disparities, as well as long-term outcomes among this and other vulnerable segments of the population. The infrastructure of FBOs affords a strong social network that reinforces positive, trusted and reciprocated relationships that are ecological in nature. Health communication programs/interventions that incorporate community input, involvement and feedback throughout the development, implementation and evaluative phases address key issues of cost and trust; factors that we suggest are highly applicable to multiple health outcomes. Our findings in a previous cancer study highlight how communicating trust and intervention strategies through strategic marketing represents one critical way that FBOs can potentially assist in reducing LBW. The DHOaD perspective suggests long-term implications of LBW and highlight LBW as a key objective for long-term public health improvements. 

Health communication must recognize the barriers imposed by social factors that increase LBW risk. Disadvantaged and segregated communities increase exposures to pollution, infectious disease (e.g., influenza), racism, gender-based violence, and stress that increase the risk of LBW infants. For example, research has suggested that increased exposure to influenza may be especially detrimental to pregnant mothers [13]. Stressors, such as threat of violence or perceived discrimination, have negative impacts on birth weight [26,27], with negative physiological responses through abnormal cortisol or dysregulation of the hypothalamic-pituitary-adrenal axis [28]. Maladaptive stress coping responses to these exposures during pregnancy such as smoking, alcohol use, and illicit substance negatively affect LBW through both fetal and maternal health (e.g., diabetes, obesity) [29]. Exposure to stressors compromise control over healthy environments, increase risk for less quality nutrition, unhealthy behaviors, and reduced prenatal health care [30,31,32] 

FBOs have the potential to address these health risks in a myriad of ways. Previous work has shown that they encourage positive health behaviors such as encouraging prayer as a beneficial stress reduction strategy [33] they offer strong social support networks; they may help with enrollment in local or federal assistance programs; provide referrals to social services (e.g., shelters); and may provide crucial information about accessing trusted local health care professionals and facilities [34]. FBOs also have the potential to address pre-maternal LBW risk factors through smoking cessation, flu vaccines, or healthy eating programs presented through sponsored health fairs. And as suggested in the findings, FBOs have the ability to communicate and market health messages via health programming/interventions, with the ability to communicate health issues, relative costs of health issues, and to foster trust in addressing these issues [35,36]. 

## 4. Conclusions 

Low socioeconomic status continues to influence LBW even when physiological pathways, behavioral antecedents, and medical solutions are identified. While FBOs may be able to increase access and utilization of health care or promote adaptive coping, more efforts will still be needed to reduce increased exposure to disadvantage and offset challenging environments. Drawing on focus group discussions with AA women, we draw parallels with health screening and suggest that community partnering in innovative health promotion approaches such as SM approaches remain necessary to overcome the barriers presented through discriminatory experiences and community-level distrust of the medical establishment among AA women. 

While enthusiastic attempts to establish DOHaD remain critical, efforts to understand the cultural and social mediation of maternal and infant health remains an issue of social inequality [10]. Additional research on the usage of DOHaD in health communication and further considerations of how community-led efforts among vulnerable populations may significantly impact behaviors remain viable solutions to address health disparities such as LBW. Clearly, more must be done to foster community engagement and trust in an effort to reduce the long-term impacts of early life conditions on lasting health inequities.

## Figures and Tables

**Table 1:** Sample Faith Based Organizations (location and number of participants per church).

Church Number	Participants (*n* = 78)	Location in the Metropolitan Area	State
Church 1	13 (Two Focus Groups)	Urban	Kansas
Church 2	14 (Two Focus Groups)	Urban Core	Kansas
Church 3	10 (Two Focus Groups)	Suburban	Missouri
Church 4	8 (Two Focus Groups)	Urban Core	Kansas
Church 5	10 (Two Focus Groups)	Urban Core	Missouri
Church 6	6 (One Focus Group)	Urban	Missouri
Church 7	3 (One Focus Group)	Suburban	Missouri
Church 8	8 (Two Focus Groups)	Urban Core	Missouri
Church 9	6 (One Focus Group)	Urban Core	Kansas
